# K13-propeller gene polymorphisms in *Plasmodium falciparum* parasite population: a systematic review protocol of burden and associated factors

**DOI:** 10.1186/s13643-018-0866-7

**Published:** 2018-11-17

**Authors:** Moses Ocan, Dickens Akena, Sam Nsobya, Moses R. Kamya, Richard Senono, Alison Annet Kinengyere, Ekwaro Obuku

**Affiliations:** 1 Department of Pharmacology & Therapeutics, Makerere University, P.O. Box 7072, Kampala, Uganda; 2 Department of Psychiatry, Makerere University, P.O. Box 7072, Kampala, Uganda; 3 Department of Medical Microbiology, Makerere University, P.O. Box 7072, Kampala, Uganda; 4 Department of Medicine, Makerere University, P.O. Box 7072, Kampala, Uganda; 5 Infectious Disease Institute, Makerere University, P. O. Box 22418, Kampala, Uganda; 6 Albert Cook library, Makerere University, P.O. Box 7072, Kampala, Uganda

**Keywords:** Artemisinin, K13-propeller gene polymorphism, Malaria, Africa, *Plasmodium falciparum*

## Abstract

**Background:**

Malaria control and prevention efforts continue to rely heavily on the use of medicines especially artemisinin agents. However, currently, the emergence of artemisinin resistance threatens this effort globally. The K13-gene polymorphisms associated with artemisinin resistance have been detected in Southeast Asia. In countries outside Southeast Asia, artemisinin resistance has not yet been confirmed.

**Methods/design:**

The articles will be obtained from the search of MEDLINE via PubMed, Scopus, EMBASE, and LILACS/VHL databases. Mesh terms will be used in the article search. Boolean operators (“AND”, “OR”) will be used in the article search. Article search will be done independently by two librarians (RS and AK). The articles will be screened for inclusion using set criteria and following the PRISMA guidelines. Data extraction will be done by two independent reviewers (NL and BB), Kappa statistic will be calculated, and any discrepancies resolved by discussion. Heterogeneity in the articles will be established using *I*^2^ statistic.

**Discussion:**

This review will focus on establishing the K13-gene polymorphisms among *Plasmodium falciparum* parasites reported from previous studies in malaria-affected countries. Artemisinin resistance has not been widely reported among parasites in Africa and other malaria-endemic countries outside Southeast Asia. However, several studies on artemisinin resistance have reported different K13-gene polymorphisms from the validated mutations found in Southeast Asia. This study will collate evidence from previous studies on the commonly reported K13 -gene polymorphisms among *P. falciparum* parasites in malaria-affected countries.

**Systematic review registration:**

PROSPERO CRD 42018084624

**Electronic supplementary material:**

The online version of this article (10.1186/s13643-018-0866-7) contains supplementary material, which is available to authorized users.

## Background

Despite the currently reported emergence and spread of resistance to artemisinin agents, there are no known effective alternative medicines for malaria treatment in the pipeline. Malaria still remains the leading cause of morbidity and mortality among children under five especially in Sub-Saharan Africa and other malaria-endemic countries [1]. Delay in the discovery of an effective alternative treatment to malaria in case of widespread artemisinin resistance threatens malaria control, treatment, and elimination efforts globally and may further worsen the consequences of malaria disease globally [2, 3]. Furthermore, widespread resistance to artemisinin agents threatens malaria control and eradication efforts [3]. This therefore calls for efforts to track and reduce the risk of global spread or the independent emergence of artemisinin resistance in other malaria-endemic countries outside Southeast Asia [4].

Artemisinin agents are the cornerstone of malaria control and eradication efforts globally. The agents are used for both uncomplicated and complicated malaria. However, in Southeast Asia, decreased parasite sensitivity to artemisinin agents associated with polymorphisms in the parasite K13-propeller gene has been reported [5, 6]. The K13-propeller gene polymorphisms associated with decreased artemisinin parasite clearance reported in Southeast Asia include C580Y, R539T, R543I, and Y493H [6]. In areas outside Southeast Asia, K13-propeller gene polymorphisms different from those detected and validated in Cambodia have been reported [5]. This has hindered regular surveillance of artemisinin resistance among *P. falciparum* parasites in other malaria-endemic countries outside Southeast Asia. Although there has been no confirmed case of artemisinin resistance outside the Southeast, the likelihood of its emergence and spread still exists. However, currently, there are no efforts to establish the phenotypic effect of the different K13-gene polymorphisms reported outside Southeast Asia on *P. falciparum* parasite response to artemisinin exposure.

Artemisinin resistance surveillance is done using both phenotypic and genotypic methods. Parasite sensitivity analysis using RSA_0-3h_ is currently used to establish parasite clearance using artemisinin agents [7, 8]. However, due to restrictive cost, there are limited sentinel sites in malaria-endemic countries where these studies are performed and are not regularly done. WHO recommends complimenting resistance surveillance by using molecular markers, as they are relatively cheaper and easier to perform [1]. The lack of confirmed molecular markers of artemisinin resistance outside Southeast Asia however has limited resistance surveillance efforts in this countries especially Africa. In malaria-affected areas outside Southeast Asia, the existence of several non-validated K13-gene polymorphisms among *Plasmodium falciparum* parasites further complicates molecular surveillance.

Currently, different K13-polymorphisms are being reported outside Southeast Asia. This could be due to variations in environmental pressures [5]. Due to variations in the reported *P. falciparum* K13-gene polymorphisms, it is currently not clear what the dominant mutations in *P. falciparum* parasites outside Southeast Asia are. In addition, the mutations are different from those that have been validated and confirmed to be associated with reduced artemisinin clearance. There has been no systematic review done to date on the K13-propeller gene polymorphisms being reported among *P. falciparum* parasites in Africa and other malaria-endemic areas outside Southeast Asia. This systematic review is thus intended to collate evidence from published articles to establish the most prevalent K13-gene polymorphism (s) in *P. falciparum* parasites in Africa and other malaria-endemic countries outside Southeast Asia.

## Methods/design

The systematic review protocol has been developed following the PRISMA-P 2015 statement [9], and this will be followed while reporting the findings of the review (Additional file 1). The review will be done following PECOS: population (P), *Plasmodium falciparum* parasites in countries affected by malaria, exposure (E), use of artemisinin agents in malaria treatment, comparator (C), none, outcome (O), K13-propeller gene polymorphisms occurring in *P. falciparum* parasites in countries affected by malaria, study design (S), the review will include articles from cross-sectional studies and those reporting using *Plasmodium falciparum* DNA samples collected on day zero (0) of clinical trial studies.

### Review question

What is the reported prevalence of K13-gene polymorphisms among *Plasmodium falciparum* parasite population in malaria-affected countries after a change in malaria treatment policy to artemisinin agents?

### Systematic review objectives

The review will meet the following objectives: (1) to establish reported prevalence of K13-propeller gene polymorphisms associated with artemisinin resistance among *P. falciparum* parasites in malaria-affected countries,, (2) to determine K13-gene polymorphisms which have been reported in *P. falciparum* parasite population in malaria-affected countries, (3) to determine factors associated with occurrence of K13-propeller gene polymorphisms associated with artemisinin resistance among *P. falciparum* parasites in malaria-affected countries.

### Registration of the protocol

The review protocol has been registered in the International Prospective Register of Systematic Reviews (PROSPERO). The current review protocol is registered with number #CRD 42018084624.

### Eligibility criteria

#### Inclusion criteria

The articles to be included in the review will be those reporting on artemisinin molecular resistance among *P. falciparum* parasites in malaria-affected countries, polymorphisms in K13-gene among *P. falciparum* confirmed through sequencing, *P. falciparum* K13-propeller gene polymorphisms, *P. falciparum* phenotypic resistance to artemisinin agents, and artemisinin resistance after change in malaria treatment policy to artemisinin agents. Articles from cross-sectional studies will be included in this review. In addition, articles reporting on *P. falciparum* parasite DNA samples collected on day zero (0) of clinical trial studies will also be included in the review.

#### Language

There will be no language restriction in the published articles.

#### Exclusion criteria

The articles will be excluded from the review if they report on *P. falciparum* parasite resistance measured using molecular markers other than K13-propeller gene polymorphisms, *P. falciparum* K13-gene polymorphisms not confirmed using sequencing methods, and resistance of *P. falciparum* parasites from stored samples collected prior to the change in malaria treatment policy to artemisinin agents.

#### Search strategy

Published articles on the prevalence of K13-propeller gene polymorphisms among *P. falciparum* parasites in malaria-affected countries will be searched from MEDLINE via PubMed, Scopus, EMBASE, LILACS/VHL, and gray literature. Article search will cover a period from 2014 to date.

#### Search terms

Boolean operators “OR”, “AND” will be used during article search. The search terms developed from reviewing articles published in peer-reviewed journals on artemisinin resistance will include artemisinin, resistance, malaria, fever, reversal, prevalence, “*Plasmodium falciparum*,” “K13-gene polymorphism,” “K13-mutation,” “resistance genes,” “resistance alleles,” “Africa,” “Sub-Saharan Africa”. The search string will be developed using the above terms. The search string used in searching for articles in PubMed is provided as Additional file 2.

#### Article search

Using the same search string, two separate searches for articles will be conducted by experienced librarians (AK and SR) from the same databases. Articles from the two independent searches will then be merged in EndNote software and duplicates removed.

#### Article review

Article search and selection process for inclusion in the review will be done following PRISMA (Preferred Reporting Items for Systematic reviews and Meta-analysis) flow. Two independent reviewers (OM and AK) will assess the articles for eligibility using a pre-set criteria, and any disagreements resolved by discussion. In case of further disagreement, this will be resolved by a final decision maker (SN).

#### Data extraction

Two independent reviewers (NL and BB) will extract data using a set extraction form. Data extraction tool in Excel spreadsheet 2007 will be used to extract information from included articles. The tool will be piloted on five (05) articles and adjusted accordingly based on findings of the pilot and the expected review outcomes (Additional file 3). Kappa agreement between the two reviewers will be calculated and any disagreement resolved by discussion. Any further disagreement between the two reviewers will be referred to the final decision maker. Strengthening Reporting of Genetic Association studies (STREGA) guidelines [10] will be used in developing a data extraction form for extracting data from the included articles.

#### Data management

Data extraction form will contain columns which capture information on study designs used, and geographical regions where primary studies were conducted. Subgroups will be created for analysis based on study designs (cross-sectional, cohort, and randomized controlled trials) and geographical regions (Southeast Asia, Africa, China, South America, and India) where primary studies reported by the articles were conducted.

#### Data analysis

Heterogeneity in the articles will be assessed using *I*^2^ statistic. The *I*^2^ statistic will be used to indicate percentage (%) heterogeneity that can be attributed to between-study variance. Interpretation is as follows: *I*^2^ = 25% (small heterogeneity), *I*^2^ = 50% (moderate heterogeneity), *I*^2^ = 75% (large heterogeneity). Narrative assessment of the data will be done. Data from articles will be pooled using measures of central tendency (means) and proportions. Additionally, a meta-analysis which is the use of statistical methods to summarize the results of independent studies [11] will be done on articles with small to moderate heterogeneity. Random effects model will be used in the analysis.

Subgroup analysis will be performed to generate aggregate/combined review outcomes. Aggregate data analysis will be done using Stata 13.0 software. The review findings will be interpreted based on the outcomes from sub-groups analysis.

#### Publication bias assessment

The asymmetry of funnel plots will be used to assess for the risk of publication bias in the included articles.

## Discussion

Artemisinin agents are an integral part of the current global efforts to control and eradicate malaria [12]. However, monitoring of resistance to these agents faces a challenge especially outside Southeast Asia due to the lack of validated molecular markers of resistance. This potentially limits the establishment of interventions to protect the efficacy of artemisinin agents [12]. Artemisinin and their derivatives are the only highly efficacious agents used currently in malaria treatment with no known alternatives [12]. This leaves malaria affected populations vulnerable to severe effects of the disease in case of widespread resistance as previously observed with chloroquine [13]. Currently, there have been reports of delayed parasite clearance by artemisinin agents in Southeast Asia, an indicator of potential emergence of resistance [6].

A study by Ariey et al [6] validated K13-propeller gene polymorphisms in *Plasmodium falciparum* parasites associated with delayed parasite clearance by artemisinin agents. Multiple studies have, however, failed to replicate identification of such molecular resistance against artemisinin agents outside Southeast Asia. In Africa, which bears the largest burden of malaria, molecular markers of artemisinin resistance have not yet been confirmed. In addition, multiple studies report K13-propeller gene polymorphisms different from those validated for causing artemisinin resistance in Southeast Asia. Thus, there is a lack of information on the role several reported K13-gene polymorphisms in malaria-endemic countries outside Southeast Asia play in artemisinin resistance. To help guide molecular artemisinin resistance surveillance outside Southeast Asia, there is a need to establish the most frequently reported K13-gene polymorphisms. In addition, validation of the role reported K13-gene polymorphisms play in parasite artemisinin resistance is urgently needed.

The current review seeks to collate evidence on K13-gene polymorphisms commonly reported in different malaria-endemic regions of the world. This will help guide in prioritizing selection of K13-gene polymorphisms outside Southeast Asia whose role in causing *Plasmodium falciparum* artemisinin resistance needs to be established. Although therapeutic efficacy studies are considered the gold standard for establishing artemisinin resistance [14], WHO recommends that therapeutic efficacy studies be supplemented by surveillance of molecular markers of resistance [15]. This is currently not possible outside Southeast Asia due to the lack of validated K13-gene polymorphisms.

## Additional files

Additional file 1: PRISMA-P 2015 Checklist. (DOC 113 kb)

Additional file 2: PUBMED SEARCH STRATEGY. (DOC 25 kb)

Additional file 3: Data Extraction form. (XLS 26 kb)

## Abbreviations

PRISMA: Preferred Reporting Items for Systematic reviews and Meta-Analysis
PROSPERO: International Prospective Register of Systematic Reviews
STREGA: Strengthening Reporting of Genetic Association studies
