# Supplementary material for: K13-propeller gene polymorphisms in Plasmodium falciparum parasite population: a systematic review protocol of burden and associated factors
Source: Syst Rev. 2018 Nov 17;7:199. doi: 10.1186/s13643-018-0866-7 (PMC6240191; doi:10.1186/s13643-018-0866-7)
Supplement: Supplementary file 1 — PRISMA-P 2015 Checklist. (DOC 113 kb) [file 13643_2018_866_MOESM1_ESM.doc]

**PRISMA-P 2015 Checklist**

# ****This checklist has been adapted for use with systematic review protocol submissions to BioMed Central journals from Table 3 in Moher D et al**:** Preferred reporting items for systematic review and meta-analysis protocols (PRISMA-P) 2015 statement. *Systematic Reviews* **2015 4:1**

# An Editorial from the Editors-in-Chief of *Systematic Reviews* details why this checklist was adapted - **Moher D, Stewart L & Shekelle P:** Implementing PRISMA-P: recommendations for prospective authors. *Systematic Reviews* 2016 **5**:15

| **Section/topic** | **#** | **Checklist item** | **Information reported** | | **Line number(s)** |
| --- | --- | --- | --- | --- | --- |
|  |  |  | **Yes** | **No** |  |
| **ADMINISTRATIVE INFORMATION** | | | | | |
| **Title** | | | | | |
| Identification | 1a | Identify the report as a protocol of a systematic review | / **Yes** |  | Lines: 02, 57, 126, 234 & 237 |
| Update | 1b | If the protocol is for an update of a previous systematic review, identify as such |  | / **No** | Not applicable |
| **Registration** | 2 | If registered, provide the name of the registry (e.g., PROSPERO) and registration number in the Abstract | / **Yes** |  | PROSPERO  #CRD42018084624 |
| **Authors** | | | | | |
| Contact | 3a | Provide name, institutional affiliation, and e-mail address of all protocol authors; provide physical mailing address of corresponding author | / **Yes** |  | Moses Ocan (MO)  Corresponding author  Department of Pharmacology & Therapeutics, Makerere University P.O. Box 7072, Kampala  [ocanmoses@gmail.com](mailto:ocanmoses@gmail.com) Dickens Akena (DA): Department of Psychiatry, Makerere University P.O. Box 7072, Kampala; [akenadickens@yahoo.co.uk](mailto:akenadickens@yahoo.co.uk) Sam Nsobya (SN): Department of Medical Microbiology, Makerere University P.O. Box 7072, Kampala  [samnsobya@yahoo.co.uk](mailto:samnsobya@yahoo.co.uk) Moses R. Kamya (MK): Department of Medicine, Makerere University P.O. Box 7072, Kampala; [**mkamya@infocom.co.ug**](mailto:mkamya@infocom.co.ug)Richard Senono (RS): Infectious Disease Institute, Makerere University P. O. Box 22418, Kampala; [rsenono@idi.co.ug](mailto:rsenono@idi.co.ug) Alison Annet Kinengyere (AK): Albert Cook library, Makerere University P.O. Box 7072, Kampala; [Alison.kine@gmail.com](mailto:Alison.kine@gmail.com)  Ekwaro Obuku (EO):  Department of Medicine, Makerere University P.O. Box 7072; [ekwaro@gmail.com](mailto:ekwaro@gmail.com) |
| Contributions | 3b | Describe contributions of protocol authors and identify the guarantor of the review | / **Yes** |  | MO drafted the initial protocol. DA, SN, MK, RS, AK, EAO edited the draft protocol. All authors reviewed the final systematic review protocol  Guarantor of review:  Prof. Moses R. Kamya |
| **Amendments** | 4 | If the protocol represents an amendment of a previously completed or published protocol, identify as such and list changes; otherwise, state plan for documenting important protocol amendments |  | / **No** | Not applicable |
| **Support** | | | | | |
| Sources | 5a | Indicate sources of financial or other support for the review | / Yes |  | NURTURE Fellowship |
| Sponsor | 5b | Provide name for the review funder and/or sponsor | / Yes |  | Grant, number D43TW010132 supported by Office of the Director, National Institutes of Health (OD), National Institute of Dental & Craniofacial Research (NIDCR), National Institute of Neurological Disorders and Stroke (NINDS), National Heart, Lung, And Blood Institute (NHLBI), Fogarty International Centre (FIC), National Institute on Minority Health and Health Disparities (NIMHD). |
| Role of sponsor/funder | 5c | Describe roles of funder(s), sponsor(s), and/or institution(s), if any, in developing the protocol | / Yes |  | The funders have had no role in the design of the study and in writing of the review protocol. |
| **INTRODUCTION** | | | | | |
| **Rationale** | 6 | Describe the rationale for the review in the context of what is already known | / Yes |  | Currently different K13-polymorphisms are being reported outside Southeast Asia. This could be due to variations in environmental pressures. Due to variations in the reported *P. falciparum* K13-gene polymorphisms, it’s currently not clear what the dominant mutations in *P. falciparum* parasites outside Southeast Asia are. In addition, the mutations are different from those that have been validated and confirmed to be associated with reduced artemisinin clearance in Southeast Asia. There has been no systematic review done to date on the K13-propeller gene polymorphisms being reported among *P. falciparum* parasites in Africa and other malaria endemic areas outside Southeast Asia. This systematic review is thus intended to collate evidence from published articles to establish the most prevalent K13-gene polymorphism (s) in *P. falciparum* parasites outside Southeast Asia. |
| **Objectives** | 7 | Provide an explicit statement of the question(s) the review will address with reference to participants, interventions, comparators, and outcomes (PICO) | / Yes |  | P: African *Plasmodium falciparum* parasite population,  E: Artemisinin agents in malaria treatment,  C: K13-propeller gene polymorphisms in Southeast Asia,  O: K13-propeller gene polymorphisms in Africa,  S: The review will include only cross-sectional studies  **Review question:**  What is the prevalence of K13-gene polymorphisms among *Plasmodium falciparum* parasite population in other malaria endemic countries compared to the *Plasmodium falciparum* parasites in Southeast Asia since the introduction of artemisinin agents in treatment of malaria? |
| **METHODS** | | | | | |
| **Eligibility criteria** | 8 | Specify the study characteristics (e.g., PICO, study design, setting, time frame) and report characteristics (e.g., years considered, language, publication status) to be used as criteria for eligibility for the review | / Yes |  | P: African *Plasmodium falciparum* parasite population,  E: Artemisinin agents in malaria treatment,  C: K13-propeller gene polymorphisms in Southeast Asia,  O: K13-propeller gene polymorphisms in Africa,  S: The review will include only cross-sectional studies  study design: Cross sectional studies  Setting: Malaria endemic countries  Time frame: One year  Years considered: 2014-to-date  language: No language restriction  Publication status: Peer reviewed journal publications |
| **Information sources** | 9 | Describe all intended information sources (e.g., electronic databases, contact with study authors, trial registers, or other grey literature sources) with planned dates of coverage | / Yes |  | Electronic databases: PubMED MEDLINE, SCOPUS, EMBASE, LILACS/VHL (All electronic database search will be done from 12-16 March 2018)  Contact with study authors: This will be used for article search (This will be done from 19-23 March 2018)  Trial registers: Not applicable in this case  Grey literature sources: Bibliography of full text articles will be searched for potential articles to be included in the review (This will be done from 26-28 March 2018) |
| **Search strategy** | 10 | Present draft of search strategy to be used for at least one electronic database, including planned limits, such that it could be repeated | / Yes |  | PUBMED search  “K13-propeller gene polymorphisms”[tiab] OR K13-polymorphisms[tiab] OR K13-gene polymorphisms[tiab] OR K13-gene[tiab] OR K13-mutation[tiab] OR K13-mutation[tiab]s OR “K13 mutation” OR mutation[Mesh] OR “K13 mutations”[tiab] OR “Resistance genes” [tiab] OR “Resistance alleles”[tiab] OR “Resistance mutations”[tiab] OR “Resistance mutation”[tiab] OR “Resistance gene”[tiab] OR “Resistance polymorphisms”[tiab] OR alleles[tiab] OR alleles[Mesh]  “Plasmodium falciparum” [tiab] OR “Plasmodium faclciparum”[Mesh] OR “plasmodium parasite” OR “plasmodium parasites” OR “malaria parasite”[tiab] “malaria parasite”[Mesh] OR “malaria parasites” OR “plasmodium falciparum malaria parasite” OR “plasmodium falciparum malaria parasites” OR malaria[tiab] OR malaria[Mesh]  Artemisinin[tiab] OR Artemisinins[Mesh] OR Artemether[tiab] OR Artesunate[tiab] OR Dihydroartemisinin[tiab] OR “Artemisinin agents”[tiab] OR ACTs[tiab]  Africa[Title/Abstract] OR “Sub-Saharan Africa”[Title/Abstract] OR “Africa South of the Sahara”[Title/Abstract] OR Algeria[Title/Abstract] OR Angola[Title/Abstract] OR Botswana[Title/Abstract] OR “Burkina Faso”[Title/Abstract] OR Burundi[Title/Abstract] OR Cameroon[Title/Abstract] OR “Cape Verde”[Title/Abstract] OR “Central African Republic”[Title/Abstract] OR Chad[Title/Abstract] OR Comoros[Title/Abstract] OR Congo[Title/Abstract] OR “Côte d'Ivoire”[Title/Abstract] OR “Ivory Coast”[Title/Abstract] OR Namibia[Title/Abstract] OR “Democratic Republic of the Congo”[Title/Abstract] OR Djibouti[Title/Abstract] OR “Equatorial Guinea”[Title/Abstract] OR Eritrea[Title/Abstract] OR Ethiopia[Title/Abstract] OR Gabon[Title/Abstract] OR Gambia[Title/Abstract] OR Ghana[Title/Abstract] OR Guinea[Title/Abstract] OR Guinea-Bissau[Title/Abstract] OR Kenya[Title/Abstract] OR Madagascar[Title/Abstract] OR Malawi[Title/Abstract] OR Malaysia[Title/Abstract] OR Mali[Title/Abstract] OR Mozambique[Title/Abstract] OR Mauritania[Title/Abstract] OR Niger[Title/Abstract] OR Nigeria[Title/Abstract] OR Rwanda[Title/Abstract] OR Senegal[Title/Abstract] OR “Sierra Leone”[Title/Abstract] OR Somalia[Title/Abstract] OR “South Africa”[Title/Abstract] OR “South Sudan”[Title/Abstract] OR Sudan[Title/Abstract] OR Swaziland[Title/Abstract] OR Togo[Title/Abstract] OR Uganda[Title/Abstract] OR “United Republic of Tanzania (Mainland)”[Title/Abstract] OR “United Republic of Tanzania (Zanzibar)”[Title/Abstract] OR Zambia[Title/Abstract] OR Zimbabwe[Title/Abstract] OR Tanzania[Title/Abstract]  Bangladesh[Title/Abstract] OR Bhutan[Title/Abstract] OR India[Title/Abstract] OR Nepal[Title/Abstract] OR “Sri Lanka”[Title/Abstract] OR China[Title/Abstract] OR “DPR Korea”[Title/Abstract] OR “Republic of Korea”[Title/Abstract] OR “Papua New Guinea”[Title/Abstract] OR “Solomon Islands”[Title/Abstract] OR Vanuatu[Title/Abstract] OR Cambodia[Title/Abstract] OR “Timor-Leste”[Title/Abstract] OR Indonesia[Title/Abstract] OR Laos[Title/Abstract] OR “Laos Peoples Democratic Republic”[Title/Abstract] OR Malaysia[Title/Abstract] OR Myanmar[Title/Abstract] OR Philippines[Title/Abstract] OR Thailand[Title/Abstract] OR Vietnam[Title/Abstract] |
| ***STUDY RECORDS*** | | | | | |
| Data management | 11a | Describe the mechanism(s) that will be used to manage records and data throughout the review | / Yes |  | Abstracted data will be kept in Excel spread sheet 2007 and articles will be kept Endnote in computers by the lead reviewer (Moses Ocan) |
| Selection process | 11b | State the process that will be used for selecting studies (e.g., two independent reviewers) through each phase of the review (i.e., screening, eligibility, and inclusion in meta-analysis) | /  Yes |  | **Article search:** Two experienced librarians (AK and SR) will independently search for the articles from established databases. Article review: Two independent reviewers will screen the articles using a pre-set criteria following PRISMA (Preferred Reporting Items for Systematic reviews and Meta-analysis) guidelines for eligibility and inclusion in the review (systematic and Meta-analysis).Any disagreement between the two reviewers will be resolved by discussion and any further disagreement referred to a tie-breaker. |
| Data collection process | 11c | Describe planned method of extracting data from reports (e.g., piloting forms, done independently, in duplicate), any processes for obtaining and confirming data from investigators | / Yes |  | Two independent reviewers will abstract data using a set abstraction form. Data abstraction tool will be developed in Excel spread sheet 2007. The tool will be piloted on five (05) articles and adjusted accordingly based on findings of the pilot and the expected review outcomes. Kappa agreement between the two reviewers will be calculated and any disagreement resolved by discussion. Any further disagreement will be referred to the tie-breaker.The authors of the reviewed articles will be contacted in case there is need for clarification and or more information needed in the article |
| **Data items** | 12 | List and define all variables for which data will be sought (e.g., PICO items, funding sources), any pre-planned data assumptions and simplifications | / Yes |  | K13-mutations, artemisinin agents used in the country of study, duration of use of artemisinin agents, Nature K13-polymorphism (synonymous of non-synonymous), source of mutation (introduced or independent emergence), Whether reported K13-mutation is associated with artemisinin resistance or Not  There is no pre-planned data assumptions and simplifications |
| **Outcomes and prioritization** | 13 | List and define all outcomes for which data will be sought, including prioritization of main and additional outcomes, with rationale | / Yes |  | Main outcome: Prevalence of K13-gene polymorphisms reported among *P. falciparum* parasites outside Southeast Asia  **Additional outcomes:** Artemisinin agents used, duration of use of artemisinin agents, country/region, year when the study was done, source of mutation, method used in identification of mutation  **Rationale:** Additional information will help provide some explanation for the main outcome of the review. The main outcome of the review is important to know as it will help guide researchers in validation of artemisinin resistance outside Southeast Asia and thus contribute to improvement of molecular surveillance of artemisinin resistance |
| **Risk of bias in individual studies** | 14 | Describe anticipated methods for assessing risk of bias of individual studies, including whether this will be done at the outcome or study level, or both; state how this information will be used in data synthesis | / Yes |  | Risk of bias in the included studies will be assessed using a risk of bias evaluation tool adopted from the STROBE (Strengthening Reporting of Observational studies in Epidemiology) guidelines.  Risk of bias will be accessed at outcome level. The analysis will be done in STATA 13.0 software  Information on quality of evidence will be used to guide selection of studies for inclusion into Meta-analysis. In addition, this information will be used in interpretation of main study outcomes and formation of sub-group analysis |
| ***DATA*** | | | | | |
| **Synthesis** | 15a | Describe criteria under which study data will be quantitatively synthesized | / Yes |  | Study data will be quantitatively synthesized only when the information is not heterogeneous across the studies |
|  | 15b | If data are appropriate for quantitative synthesis, describe planned summary measures, methods of handling data, and methods of combining data from studies, including any planned exploration of consistency (e.g., *I* ^2^, Kendall’s tau) | / Yes |  | Heterogeneity in the data will be assessed using I^2^-test, forest plots will be drowned. Random effects analysis will used. |
|  | 15c | Describe any proposed additional analyses (e.g., sensitivity or subgroup analyses, meta-regression) | / Yes |  | Sub-group analysis will be done (countries/regions, duration of use of artemisinin agents, study dseign) |
|  | 15d | If quantitative synthesis is not appropriate, describe the type of summary planned | / Yes |  | Proportions/frequencies |
| **Meta-bias(es)** | 16 | Specify any planned assessment of meta-bias(es) (e.g., publication bias across studies, selective reporting within studies) | / Yes |  | Risk of bias in the included studies will be assessed using a risk of bias evaluation tool adopted from the STROBE (Strengthening Reporting of Observational studies in Epidemiology) guidelines |
| **Confidence in cumulative evidence** | 17 | Describe how the strength of the body of evidence will be assessed (e.g., GRADE) | / Yes |  | Quality of evidence of each outcome will be assessed following a GRADE system. This will be based on an eight criterion system which includes, assessment of; risk of bias, indirectness, inconsistency, imprecision, and publication bias. The quality of evidence for each outcome will be graded as HIGH, MODERATE, LOW or VERY LOW. Grading of quality of evidence will be done by two independent authors using GRADEpro software. Decisions to down grade the quality of evidence of each outcome will be made after assessment of whether any identified limitation (s) represent a serious threat (in which case it is downgraded by 1 level) or a very serious threat (downgraded by 2 levels) to the validity of overall review results. The decision to upgrade the quality of evidence of the outcome after an initial low rating will be made if, there is a large magnitude of effect (strong association), dose response, and when the effect of all plausible confounding factors would be to reduce the effect or suggest a spurious effect. |
