# Supplementary material for: K13-propeller gene polymorphisms in Plasmodium falciparum parasite population: a systematic review protocol of burden and associated factors
Source: Syst Rev. 2018 Nov 17;7:199. doi: 10.1186/s13643-018-0866-7 (PMC6240191; doi:10.1186/s13643-018-0866-7)
Supplement: Supplementary file 2 — PUBMED SEARCH STRATEGY. (DOC 25 kb) [file 13643_2018_866_MOESM2_ESM.doc]

**PUBMED SEARCH STRATEGY**

“K13-propeller gene polymorphisms”[tiab] OR K13-polymorphisms[tiab] OR K13-gene polymorphisms[tiab] OR K13-gene[tiab] OR K13-mutation[tiab] OR K13-mutation[tiab]s OR “K13 mutation” OR mutation[Mesh] OR “K13 mutations”[tiab] OR “Resistance genes” [tiab] OR “Resistance alleles”[tiab] OR “Resistance mutations”[tiab] OR “Resistance mutation”[tiab] OR “Resistance gene”[tiab] OR “Resistance polymorphisms”[tiab] OR alleles[tiab] OR alleles[Mesh]

“Plasmodium falciparum” [tiab] OR “Plasmodium faclciparum”[Mesh] OR “plasmodium parasite” OR “plasmodium parasites” OR “malaria parasite”[tiab] “malaria parasite”[Mesh] OR “malaria parasites” OR “plasmodium falciparum malaria parasite” OR “plasmodium falciparum malaria parasites” OR malaria[tiab] OR malaria[Mesh]

Artemisinin[tiab] OR Artemisinins[Mesh] OR Artemether[tiab] OR Artesunate[tiab] OR Dihydroartemisinin[tiab] OR “Artemisinin agents”[tiab] OR ACTs[tiab]

Africa[Title/Abstract] OR “Sub-Saharan Africa”[Title/Abstract] OR “Africa South of the Sahara”[Title/Abstract] OR Algeria[Title/Abstract] OR Angola[Title/Abstract] OR Botswana[Title/Abstract] OR “Burkina Faso”[Title/Abstract] OR Burundi[Title/Abstract] OR Cameroon[Title/Abstract] OR “Cape Verde”[Title/Abstract] OR “Central African Republic”[Title/Abstract] OR Chad[Title/Abstract] OR Comoros[Title/Abstract] OR Congo[Title/Abstract] OR “Côte d'Ivoire”[Title/Abstract] OR “Ivory Coast”[Title/Abstract] OR Namibia[Title/Abstract] OR “Democratic Republic of the Congo”[Title/Abstract] OR Djibouti[Title/Abstract] OR “Equatorial Guinea”[Title/Abstract] OR Eritrea[Title/Abstract] OR Ethiopia[Title/Abstract] OR Gabon[Title/Abstract] OR Gambia[Title/Abstract] OR Ghana[Title/Abstract] OR Guinea[Title/Abstract] OR Guinea-Bissau[Title/Abstract] OR Kenya[Title/Abstract] OR Madagascar[Title/Abstract] OR Malawi[Title/Abstract] OR Malaysia[Title/Abstract] OR Mali[Title/Abstract] OR Mozambique[Title/Abstract] OR Mauritania[Title/Abstract] OR Niger[Title/Abstract] OR Nigeria[Title/Abstract] OR Rwanda[Title/Abstract] OR Senegal[Title/Abstract] OR “Sierra Leone”[Title/Abstract] OR Somalia[Title/Abstract] OR “South Africa”[Title/Abstract] OR “South Sudan”[Title/Abstract] OR Sudan[Title/Abstract] OR Swaziland[Title/Abstract] OR Togo[Title/Abstract] OR Uganda[Title/Abstract] OR “United Republic of Tanzania (Mainland)”[Title/Abstract] OR “United Republic of Tanzania (Zanzibar)”[Title/Abstract] OR Zambia[Title/Abstract] OR Zimbabwe[Title/Abstract] OR Tanzania[Title/Abstract]

Bangladesh[Title/Abstract] OR Bhutan[Title/Abstract] OR India[Title/Abstract] OR Nepal[Title/Abstract] OR “Sri Lanka”[Title/Abstract] OR China[Title/Abstract] OR “DPR Korea”[Title/Abstract] OR “Republic of Korea”[Title/Abstract] OR “Papua New Guinea”[Title/Abstract] OR “Solomon Islands”[Title/Abstract] OR Vanuatu[Title/Abstract] OR Cambodia[Title/Abstract] OR “Timor-Leste”[Title/Abstract] OR Indonesia[Title/Abstract] OR Laos[Title/Abstract] OR “Laos Peoples Democratic Republic”[Title/Abstract] OR Malaysia[Title/Abstract] OR Myanmar[Title/Abstract] OR Philippines[Title/Abstract] OR Thailand[Title/Abstract] OR Vietnam[Title/Abstract]
